# Sharing of Diverse Mycorrhizal and Root-Endophytic Fungi among Plant Species in an Oak-Dominated Cool–Temperate Forest

**DOI:** 10.1371/journal.pone.0078248

**Published:** 2013-10-21

**Authors:** Hirokazu Toju, Satoshi Yamamoto, Hirotoshi Sato, Akifumi S. Tanabe

**Affiliations:** 1 Graduate School of Global Environmental Studies, Kyoto University, Sakyo, Kyoto, Japan; 2 Graduate School of Human and Environmental Studies, Kyoto University, Sakyo, Kyoto, Japan; 3 National Research Institute of Fisheries Science, Fisheries Research Agency, Fukuura, Kanazawa, Yokohama, Kanagawa, Japan; Emory University, United States of America

## Abstract

Most terrestrial plants interact with diverse clades of mycorrhizal and root-endophytic fungi in their roots. Through belowground plant–fungal interactions, dominant plants can benefit by interacting with host-specific mutualistic fungi and proliferate in a community based on positive plant–mutualistic fungal feedback. On the other hand, subordinate plant species may persist in the community by sharing other sets (functional groups) of fungal symbionts with each other. Therefore, revealing how diverse clades of root-associated fungi are differentially hosted by dominant and subordinate plant species is essential for understanding plant community structure and dynamics. Based on 454-pyrosequencing, we determined the community composition of root-associated fungi on 36 co-occurring plant species in an oak-dominated forest in northern Japan and statistically evaluated the host preference phenotypes of diverse mycorrhizal and root-endophytic fungi. An analysis of 278 fungal taxa indicated that an ectomycorrhizal basidiomycete fungus in the genus *Lactarius* and a possibly endophytic ascomycete fungus in the order Helotiales significantly favored the dominant oak (*Quercus*) species. In contrast, arbuscular mycorrhizal fungi were generally shared among subordinate plant species. Although fungi with host preferences contributed to the compartmentalization of belowground plant–fungal associations, diverse clades of ectomycorrhizal fungi and possible root endophytes were associated not only with the dominant *Quercus* but also with the remaining plant species. Our findings suggest that dominant-ectomycorrhizal and subordinate plant species can host different subsets of root-associated fungi, and diverse clades of generalist fungi can counterbalance the compartmentalization of plant–fungal associations. Such insights into the overall structure of belowground plant–fungal associations will help us understand the mechanisms that facilitate the coexistence of plant species in natural communities.

## Introduction

 In terrestrial ecosystems, plants interact with various types of mutualistic animals and microbes, and plant community dynamics depend on the nature of these plant–partner interactions [1-3]. Insect and avian pollinators, for example, are essential for sexual reproduction in diverse plant species in various types of terrestrial ecosystems [4,5]. Although plant species in a community compete with each other for light or space, plant–pollinator interactions can offset such competitive plant-to-plant interactions if co-occurring plant species collectively pay the cost of supporting populations of generalist pollinators [6,7]. The dependence of plant community dynamics on plant–partner interactions is also expected in plant–seed disperser systems [8-10], and is considered one of the major determinants of local plant community structure [7,11]. 

 Although plant–animal interactions are prevalent in natural forests and grasslands, another ubiquitous plant–partner interaction exists that has great potential to impact plant community dynamics: belowground associations between plants and root-associated fungi [12-14]. Since the early stage of land colonization 460 million years ago, most terrestrial plants have hosted mycorrhizal fungal symbionts in their roots [15-17]. These mycorrhizal fungi provide host plants with soil nutrients and water, thereby increasing the growth or survival rates of their hosts [12,18,19]. In addition to mycorrhizal fungi, plant roots are colonized by various clades of endophytic fungi [20-22]. Although many of these fungi are regarded as commensalistic symbionts, recent studies have shown that they can benefit their hosts by mineralizing soil nutrients in the rhizosphere or protecting hosts from soil pathogens [21,23]. Because the sharing of root-associated fungi could facilitate the coexistence of plant species [24,25], studies that clarify how diverse clades of root-associated fungi are shared within a plant community are essential to our understanding of plant community dynamics and stability. 

 In examining the overall structure of belowground plant–fungal associations, the host preferences of fungi are critical for evaluating how diverse functional groups of fungi differentially associate with plant communities. Ectomycorrhizal fungi, for example, are known to interact with plants in several families, including Fagaceae, Betulaceae, Dipterocarpaceae, Caesalpiniaceae, and Pinaceae [26-29]. These ectomycorrhizal plants are dominant in a broad range of temperate and tropical forests [30-32]. Hence, ectomycorrhizal fungi have been hypothesized to facilitate the dominance of their host plants by specifically supporting the growth or survival of particular ectomycorrhizal host species [13,30,33]. For example, an ectomycorrhizal Caesalpiniaceae species (*Dicymbe corymbosa*) forms large dominant patches within a tropical rain forest, wherein 98% of surveyed plant species host arbuscular mycorrhizal fungi but not ectomycorrhizal fungi [33]. Within the patches, associations with ectomycorrhizal fungi increase the survival rate of the seedlings of the ectomycorrhizal dominant plant [33]. Thus, increase in the population density of the ectomycorrhizal plant may increase the relevance of its specific ectomycorrhizal fungi within the patches (i.e., positive plant–ectomycorrhizal-fungal feedback), thereby promoting the dominance of the ectomycorrhizal plant [33]. In contrast to ectomycorrhizal fungi, many arbuscular mycorrhizal and root-endophytic fungi are believed to associate with plant species in diverse families [34-37]. Therefore, these fungi may benefit diverse clades of subordinate plant species in the forests that are dominated by ectomycorrhizal plant species. Consequently, by collectively supporting arbuscular-mycorrhizal fungal populations, those plant species may be able to counteract the positive feedbacks formed by dominant-ectomycorrhizal plants and their specific fungi. For the first step to examine this potential mechanism of plant species coexistence, we need to evaluate how dominant ectomycorrhizal and subordinate plant species host diverse root-associated fungi within a forest. 

 In a cool-temperate forest in northern Japan, we tested the hypothesis that subordinate plant species share the fungal species that are not common to dominant-ectomycorrhizal plant species. We first determined the root-associated fungal communities on 36 co-occurring plant species and statistically evaluated the host preferences of ectomycorrhizal, arbuscular mycorrhizal, and endophytic fungal symbionts. In the forest, two fagaceous species (*Quercus crispula* and *Q. dentata*) and their hybrids are dominant, while most of the remaining plant species are possibly arbuscular mycorrhizal. Thus, this system provided an opportunity to examine how belowground fungal communities differ between dominant ectomycorrhizal and subordinate plant species. Based on massively parallel pyrosequencing of fungal internal transcribed spacer (ITS) sequences, we conducted an extensive community survey of fungal symbionts in the roots of 36 plant species. The resulting fungal community data set allowed us to infer how plant species shared diverse functional groups of root-associated fungi in an oak-dominated forest. Moreover, we used the data set to evaluate the degree of each fungus’ host preference. To date, many mycological studies have evaluated the compatibility of plant–fungal symbiosis by inoculating individual fungal species onto plant species in conditioned experimental environments [35,38,39]. While these cross-inoculation experiments provide invaluable information about the “potential” or “fundamental” host ranges of fungi, plant–fungal associations that are “realized” in natural environments should depend on the composition of the local plant community [32,40] and/or abiotic soil conditions [41,42]. To evaluate the host preference phenotypes that are realized in a local forest (hereafter, local host preference), we used statistical indices [43-45] that measure how the host range of an individual fungus deviates from the expected pattern in random plant–fungal associations. 

## Materials and Methods

### Sampling and DNA extraction

 Roots were sampled from a cool–temperate forest in the Tomakomai Experimental Forest of Hokkaido University, Tomakomai, Hokkaido, Japan (42°40'N, 141°36'E; parent material = volcanic ash), from August 9 to 11, 2011. At the study site, two deciduous oak species (*Q. crispula* and *Q. dentata*) and their hybrids are dominant ([46]; hereafter, *Quercus* spp.), while maples (*Acer* spp.) and broad-leaved shrubs (e.g., *Pachysandra terminalis*) co-occur. A 30 × 30-m plot was established and sampling positions were set at 1-m intervals. Samples were collected from 961 sampling positions (31 rows × 31 columns), although the last sample was not applied so that the following high-throughput polymerase chain reaction (PCR) protocol with 96-well plates could be used. At each sampling position, we haphazardly sampled two segments of terminal root (approximately 2 cm) from the upper part of the A horizon (3 cm below the soil surface). Terminal root samples were collected indiscriminately regarding root morphology or apparent mycorrhizal type; therefore, the samples as a whole should represent the relative frequency of plant–fungal associations in the horizon at the study site [22,47,48]. This sampling strategy also helped us describe the composition of the belowground plant community, which potentially affected the local host preference of root-associated fungi. The root samples were immediately preserved in absolute ethanol and stored at -25°C in the laboratory. All necessary permits for the sample collection were issued by the Tomakomai Experimental Forest of Hokkaido University, Hokkaido, Japan. 

### DNA extraction, PCR, and pyrosequencing

 One 2-cm terminal root was randomly selected from each of the 960 sampling positions. To remove all soil from each sample, it was placed in 70% ethanol with 1-mm zirconium balls and shaken 15 times/s for 2 min using a TissueLyser II (Qiagen, Venlo, The Netherlands). This procedure removed soil from the terminal root samples [22]. The washed roots were frozen at -25°C and then pulverized by shaking with 4-mm zirconium balls 20 times/s for 3 min using a TissueLyser II. Plant and fungal DNA were extracted from each root sample by a cetyl trimethyl ammonium bromide (CTAB) method described elsewhere [49].

 We sequenced host-plant chloroplast *rbcL* and fungal ITS sequences based on a tag-encoded massively parallel pyrosequencing analysis [22]. For each root sample, a 0.5-kb *rbcL* gene fragment was amplified using the forward primer rbcL_F3 (5'-AAY TCC CAA CCA TTY ATG CG-3') fused with 454 pyrosequencing Adaptor A (5'-CCA TCT CAT CCC TGC GTG TCT CCG ACT CAG-3') and the 8-mer molecular ID [50] of each sample, and the reverse primer rbcL_R4 (5'-CAT ATG CCA AAC RTG AAT ACC-3') fused with 454 Adaptor B (5'-CCT ATC CCC TGT GTG CCT TGG CAG TCT CAG-3'). PCR was conducted using a temperature profile of 95°C for 10 min, followed by 40 cycles at 94°C for 20 s, 56°C for 30 s, 72°C for 90 s and a final extension at 72°C for 7 min using an Ampdirect Plus buffer system (Shimadzu Corp., Kyoto, Japan) and BIOTAQ HS DNA Polymerase (Bioline, London, UK). 

 To analyze the fungal ITS sequences, the entire ITS region and the partial ribosomal large subunit region was amplified using the fungus-specific high-coverage primer ITS1F_KYO2 [51] and the universal primer LR3 (http://www.biology.duke.edu/fungi/mycolab/primers.htm). PCR was conducted using a temperature profile of 95°C for 10 min, followed by 20 cycles at 94°C for 20 s, 50°C for 30 s, 72°C for 120 s, and a final extension at 72°C for 7 min using an Ampdirect Plus buffer system and BIOTAQ HS DNA Polymerase (Shimadzu). The PCR product from each root sample was subjected to a second PCR step that targeted the ITS2 region. The second PCR was conducted using the universal primer ITS3_KYO2 [51] fused with 454 Adaptor A and each sample-specific molecular ID, and the reverse universal primer LR_KYO1b (5'-MGC WGC ATT CCC AAA CWA-3') fused with 454 Adaptor B. A buffer system of Taq DNA Polymerase with Standard Taq Buffer (New England BioLabs, Ipswich, MA, USA) was used with a temperature profile of 95°C for 1 min, followed by 40 cycles at 94°C for 20 s, 50°C for 30 s, 72°C for 60 s, and a final extension at 72°C for 7 min. 

 The *rbcL* and ITS amplicons were subjected to pyrosequencing. Due to the large sample size, the first 480 and the remaining 480 samples were sequenced separately using a GS Junior sequencer (Roche, Basel, Switzerland). The *rbcL* and ITS amplicons from the first 480 root samples were pooled and purified using ExoSAP-IT (GE Healthcare, Little Chalfont, Buckinghamshire, UK) and a QIAquick PCR Purification Kit (Qiagen). The sequencing of the first 480 samples was conducted according to the manufacturer’s instructions. The amplicons of the remaining 480 samples were pooled and purified, and then sequenced in a second run. 

### Assembling of pyrosequencing reads

 Using a GS Junior sequencer, 103,233 and 130,305 reads were obtained for the first and second runs, respectively. The full dataset of the runs was deposited on the Sequence Read Archive of DNA Data Bank of Japan (accession: DRA000964). For the pyrosequencing reads, the trimming of low-quality 3’ tails was conducted with a minimum quality value of 27 [52]. After the trimming step, 87,635 (33,716 *rbcL* and 53,919 ITS reads) and 101,366 (36,660 *rbcL* and 64,706 ITS reads) reads for the first and second runs, respectively, passed the filtering process in which *rbcL* reads that were shorter than 400 bp and ITS reads with fewer than 150 bp, excluding the forward primer, molecular ID, and ribosomal large subunit positions, were discarded. *RbcL* and ITS reads were recognized by their primer position sequences and analyzed separately. For each gene, pyrosequencing reads were sorted based on combinations of the sample-specific molecular ID and the pyrosequencing run. The molecular ID and forward primer sequences were removed before the assembly process. De-noising of sequencing data was performed based on the assembly analysis detailed below (cf. [53]).

 For the analysis of the host plant *rbcL* gene, reads were assembled using Assams-assembler v0.1.2013.01.01 [54], which is a highly parallelized extension of the Minimus assembly pipeline [55]. Reads in each sample were assembled with a minimum cutoff similarity of 97% to remove pyrosequencing errors, and the consensus *rbcL* gene sequence of each root sample was then obtained. After eliminating possible chimeras using UCHIME v4.2.40 [56], with a minimum score of 0.3 to report a chimera, the consensus sequences for the root samples (within-sample consensus sequences) were further assembled across samples with a minimum similarity setting of 99.8%. These consensus sequences (among-sample consensus sequences) were compared to reference *rbcL* sequences in the NCBI nucleotide database (http://www.ncbi.nlm.nih.gov/) to identify the host plant species of each root sample. Due to high variance in the number of obtained sequencing reads for the *rbcL* gene (mean = 73.3, SD = 78.5, *N* = 960), the number of samples with host-plant information was 635. 

 In the analysis of the fungal ITS2 region, 118,625 reads (53,919 from the first run and 64,706 from the second run) were subjected to the detection and removal of chimeras using UCHIME after within-sample consensus sequences with a minimum cutoff similarity of 97% were obtained. Of the 118,625 ITS reads, 648 reads were discarded as chimeras, leaving a total of 117,977 reads.

 Within-sample consensus sequences for the 117,977 reads were assembled across samples. Given that fungal ITS sequences generally show up to 5% intraspecific variation [57], the minimum cutoff similarity for the among-sample assembling process was set to 95% in Assams-assembler. The resulting consensus sequences represented fungal operational taxonomic units (OTUs; Appendix S1). Of the 117,977 reads, 341 were excluded as singletons. Samples with fewer than 20 high-quality reads were eliminated, leaving 876 root samples. On average, 133.9 (SD = 66.0) ITS reads were obtained for each sample and the mean number of OTUs per sample was 8.0 (SD = 4.4; S1a). Because sequences of rare OTUs are likely to contain a high proportion of pyrosequencing errors, OTUs that consisted of less than five reads were excluded from the following analysis. Consequently, after the ITS and the abovementioned *rbcL* data were combined, both symbiont and host information was available for 577 samples. 

### Molecular identification of fungi

 To infer the taxonomy of respective OTUs systematically, local BLAST databases were prepared based on the "nt" database downloaded from the NCBI ftp server (http://www.ncbi.nlm.nih.gov/Ftp/) on November 18, 2012. Molecular identification of OTUs was conducted through local BLAST searches using Claident v0.1.2012.11.23 [22,58], which integrated BLAST+ [59] and NCBI taxonomy-based sequence identification engines based on the lowest common ancestor algorithm [60]. Based on the molecular identification, OTUs were classified into ectomycorrhizal fungi, arbuscular mycorrhizal fungi, and fungi with unknown ecological functions. To screen for ectomycorrhizal fungi, we referred to a review by Tedersoo et al. [29]: OTUs in the genera and/or families that were predominantly ectomycorrhizal were classified as putative ectomycorrhizal fungi.

### Community data matrices

 For each of the 577 samples from which both *rbcL* and ITS sequences were successfully obtained, the presence/absence of respective fungal OTUs was evaluated using the following process. To reduce the variance in α-diversity among samples that results from variance in sequencing effort (i.e., variance in the number of sequencing reads among samples), only OTUs with more than 5% of sample total reads were regarded as being present in a sample (Appendix S2). Through this process, a binary matrix that depicted the presence or absence of OTUs in each sample was created (Appendix S3; hereafter, “sample-level” matrix). 

 The “sample-level” matrix was used to construct a matrix that represented associations between plant species and fungal OTUs (Appendix S4: hereafter, “plant x fungal” matrix). In the “plantx fungal” matrix, rows represented plant species and columns represented fungal OTUs, and the value in a cell represented the number of root samples in which the focal plant–fungal association was observed (Appendix S4). 

### Fungal diversity and composition of plant–fungal associations

 The taxonomic diversity of root-associated fungi in the study site was first evaluated by the number of OTUs that belonged to each taxon at the phylum, order, or genus level. We then evaluated the composition of belowground plant–fungal associations by weighting the occurrence of each fungal OTU with the number of root samples in which the focal OTU was observed using the “plant x fungal” matrix (Appendix S4). The weighted composition of root-associated fungi was compared between the dominant plants, *Quercus* spp. (i.e., *Q. crispula*, *Q. dentata*, and their hybrids), and the remaining plant species (subordinate plant species) at each of the phylum, order, and genus level by a chi-square test.

### Number of fungal OTUs shared between plant species

 Based on the “plant x fungal” matrix, the number of fungal OTUs shared between host species was calculated for each pair of plant species. In addition to the total number of fungal OTUs, the numbers of ectomycorrhizal fungal OTUs and arbuscular mycorrhizal OTUs were calculated for each pair of plant species. 

### Local host preference analysis

 We statistically screened for fungal OTUs that preferentially colonized *Quercus* roots and OTUs that preferred the roots of subordinate plant species in the community. Based on the multinomial species classification method (CLAM; [45]), fungal OTUs were classified into the following categories: fungi preferring *Quercus* spp., fungi preferring subordinate plants, fungi common on both *Quercus* and subordinate plants, and fungi that were too rare to be assigned a host preference. The CLAM analysis was performed based on the “sample-level” data matrix (Appendix S3) using the vegan v.2.0-2 package [61] of R (http://cran.r-project.org/) with the “supermajority” rule [45]. The CLAM test was also used to compare fungal community structure between *Quercus* spp. and commonly-observed two *Acer* species (*A. mono* and *A*. sp. 1; hereafter, common *Acer* spp.; see results) and between common *Acer* spp. and the remaining subordinate plants (i.e., plant species other than *Quercus* spp. and common *Acer* spp.). 

 To evaluate the local host preference of each fungal OTU in the cool–temperate forest, we estimated the *d’* index of the specialization of interspecific interactions [43,44] based on the “plant x fungal” matrix (Appendix S4). The *d’* index measures how strongly a fungus deviates from a random choice among plant partners that are available at a study site. The index is derived from Shannon’s diversity index (Shannon’s entropy), which is commonly used in community ecology: the *d’* index is standardized to range from 0 (extreme generalization) to 1 (extreme specialization) [43,44]. The “bipartite” v1.17 package [62] of R was used to estimate *d’* for each fungal OTU. Observed *d’* index values were compared with values from a randomized “plant x fungal” matrix, in which combinations of plant species and fungal OTUs were randomized with the “vaznull” model [63] using the bipartite package (10,000 permutations). A *d’* index value that is higher than would be expected by chance indicates a preference for a host-plant species in a fungal OTU. 

## Results

### Plant and fungal diversity

 Sequencing of the chloroplast *rbcL* gene revealed that the 577 terminal root samples represented 36 plant species (Figure S1b): note that *Quercus* spp. is counted as one species in the calculation. Among them, *Quercus* spp. were the most common (44.2% [255/577]), while two *Acer* species (*A.* mono and *A*. sp. 1) represented 17.5% (101/577) of the root samples. Various types of plant species such as deciduous broad-leaved trees (e.g., *Prunus*, and *Ulmus*), evergreen conifers (*Picea*), shrubs (e.g., *Pachysandra* and *Spiraea*), woody vines (*Schisandra*), and herbaceous species (e.g., *Maianthemum* and *Carex*) were also observed. 

 From the 577 sequenced terminal-root samples, we obtained 278 OTUs, excluding possible chimeras, non-fungal sequences, and OTUs that had fewer than five pyrosequencing reads (Appendix S2). Of the 278 OTUs observed, 120 (43.2%) were ascomycetes, 127 (45.7%) were basidiomycetes, 21 (0.6%) were glomeromycetes, one (0.4%) was a chytridiomycete, and nine (3.2%) were unidentified at the phylum level (Figure S2a). At the order level, Helotiales (11.2%), Agaricales (12.6%), Russulales (8.3%), Thelophorales (7.6%), and Glomerales (6.1%) were the most common (Figure S2b). At the genus level, three ectomycorrhizal genera, *Russula* (6.5%), *Tomentella* (5.8%), and *Sebacina* (4.3%), were the most common, while diverse ectomycorrhizal (e.g., *Inocybe*, *Cortinarius*, *Lactarius*, *Clavulina*, *Tuber*, and *Cenococcum*), arbuscular mycorrhizal (e.g., *Glomus* and *Rhizophagus*), and non-mycorrhizal (e.g., *Mycena*, *Cladophialophora*, *Scleropezicula*, *Trichosporon*, and *Mortierella*) genera were also detected (Figure S2c).

### Composition of plant–fungal associations

**Figure 1:**
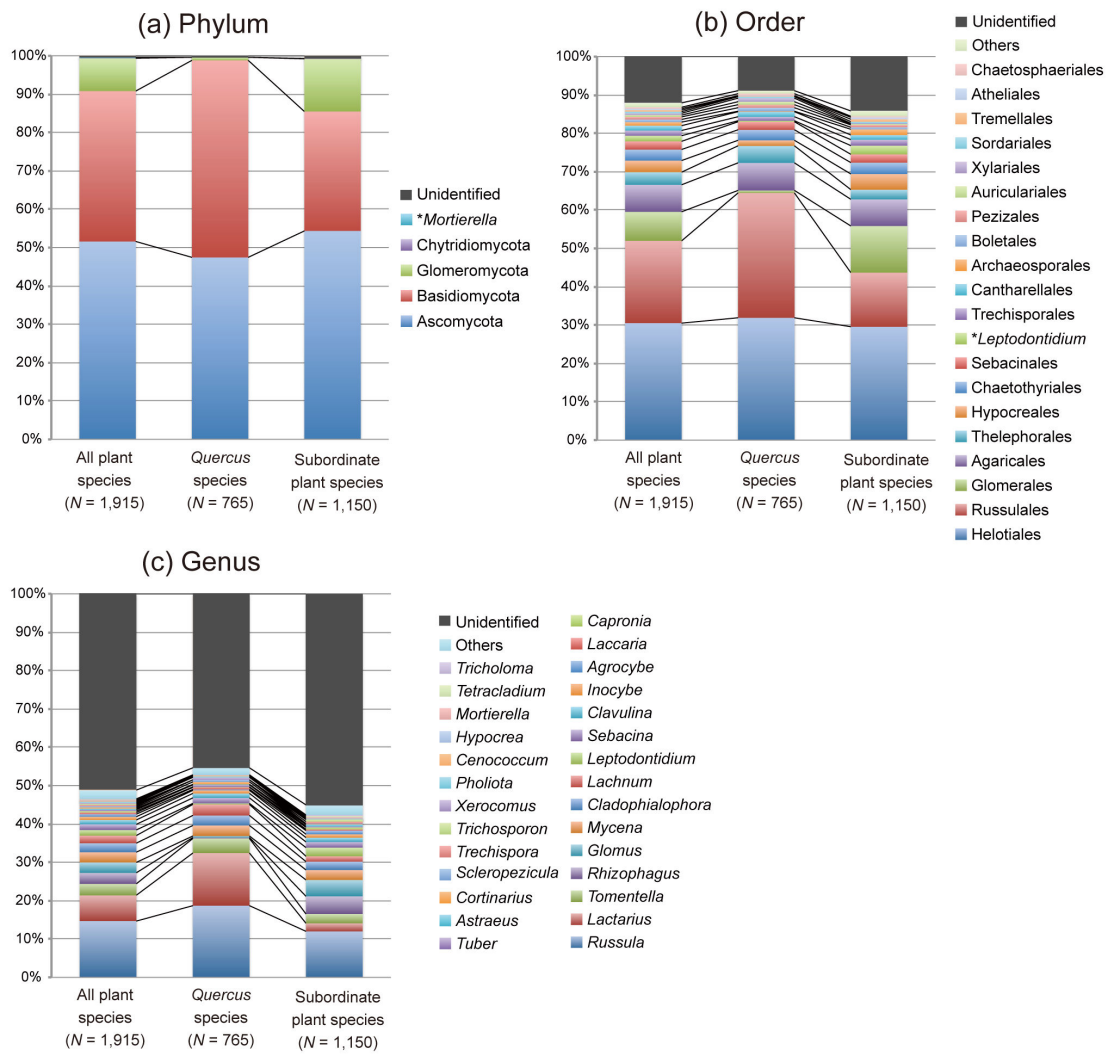
Composition of the plant–fungal associations observed at the study site. The fungal-OTU composition (Figure S2) was weighted by the number of root samples from which a fungal OTU was observed at each of the phylum (a), order (b) and genus (c) levels. In total, 1,915, 765 and 1,150 plant–fungal associations were observed for all the 36 plant species (left), *Quercus* species (middle) and subordinate plant species (right), respectively. OTUs whose taxonomy is unsettled at the phylum or order level are indicated by asterisk.

 When the composition of belowground plant–fungal associations was weighted by the number of root samples in which a fungal OTU was detected (Figure 1), Helotiales and Russulales fungi accounted for more than half of the observations. In addition to these two orders, fungi in Glomerales and Agaricales were commonly observed in the root samples. At the genus level, the three ectomycorrhizal genera, *Russula*, *Lactarius*, and *Tomentella*, accounted for one-quarter of the plant–fungal associations (Figure 1).

 Further analysis revealed that the weighted community composition of root-associated fungi differed significantly among the dominant plants, *Quercus* spp., and other plant species at each of the phylum (χ^2^ = 143.7, df = 4, *P* < 0.0001), order (χ^2^ = 216.8, df = 30, *P* < 0.0001), and genus (χ^2^ = 265.0, df = 61, *P* < 0.0001) levels. At the phylum level, associations with basidiomycete fungi were the most common for *Quercus* spp. (51.4%), while associations with ascomycete fungi were the most common for subordinate plant species (54.3%; Figure 1). Moreover, only 0.8% of *Quercus*–fungal associations involved glomeromycete (arbuscular mycorrhizal) fungi, while this fungal taxon accounted for 13.7% of the plant–fungal associations for subordinate plant species (Figure 1). At the order level, 32.5% of *Quercus* spp. associations involved Russulales fungi, while fungi in this order accounted for only 14.1% of the associations for subordinate plant species (Figure 1). In contrast to these taxa, the proportion of Helotiales fungi was comparable between *Quercus* (31.9%) and subordinate plant (29.6%) species (Figure 1). At the genus level, *Quercus* species harbored a higher proportion of ectomycorrhizal lineages, such as *Russula* (18.7%) and *Lactarius* (13.7%) compared to subordinate plant species (Figure 1). Of the two ectomycorrhizal basidiomycete genera, *Russula* was observed on non-*Quercus* hosts at a relatively high frequency (12.0%), while *Lactarius* was rare on plant species other than *Quercus* (2.1%; Figure 1). 

### Number of fungal OTUs shared between plant species

 At the study site, each plant species shared at least one root-associated fungal symbiont with other plant species (Figure S3). In particular, 50, 49, and 34 fungal OTUs were shared between *Quercus* species. and *Acer mono*, *Acer* sp. 1, and *Schisandra chinensis*, respectively (Figure S3). 

**Figure 2:**
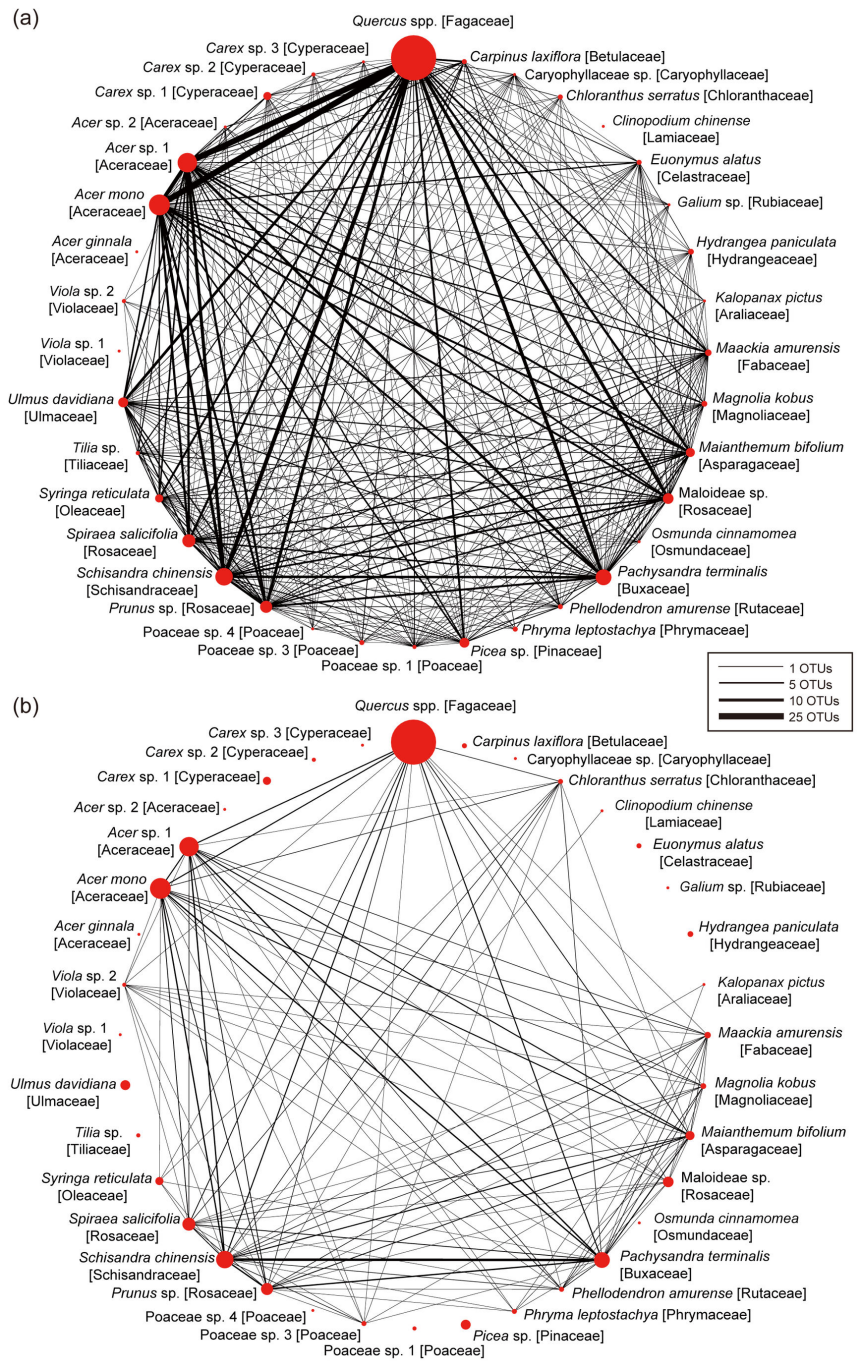
Sharing of fungal OTUs among plant species in the community. (a) Number of ectomycorrhizal fungal OTUs shared among plant species. The line thickness is proportional to the number of fungal OTUs shared between each pair of plant species. The size of circles roughly represents the composition of plant species in the samples (Figure S1B). (b) Number of arbuscular mycorrhizal fungal OTUs shared among plant species.

 Our results also showed that many ectomycorrhizal OTUs colonized not only the dominant *Quercus* species but also the remaining plant species in the community (Figure 2a). For example, 16,24,25 ectomycorrhizal fungal OTUs were shared between *Quercus* spp. and *Acer mono*, *Acer* sp. 1, and *S. chinensis*, respectively (Figure 2a). We also found that a maximum of four arbuscular mycorrhizal fungal OTUs were shared between *Quercus* species and each subordinate plant species (Figure 2b). In contrast, arbuscular mycorrhizal fungal OTUs were shared among various deciduous trees (e.g., *Acer mono*, *Acer* sp. 1, and *Magnolia kobus*), shrubs (e.g., *P. terminalis* and *Spiraea salicifolia*), woody vines (e.g., *S. chinensis*) and herbaceous plants (e.g., *Maianthemum bifolium*) in the forest. For example, 10 arbuscular mycorrhizal fungal OTUs were shared between *P. terminalis* and *S. chinensis.*

### Local host preference analysis

**Figure 3:**
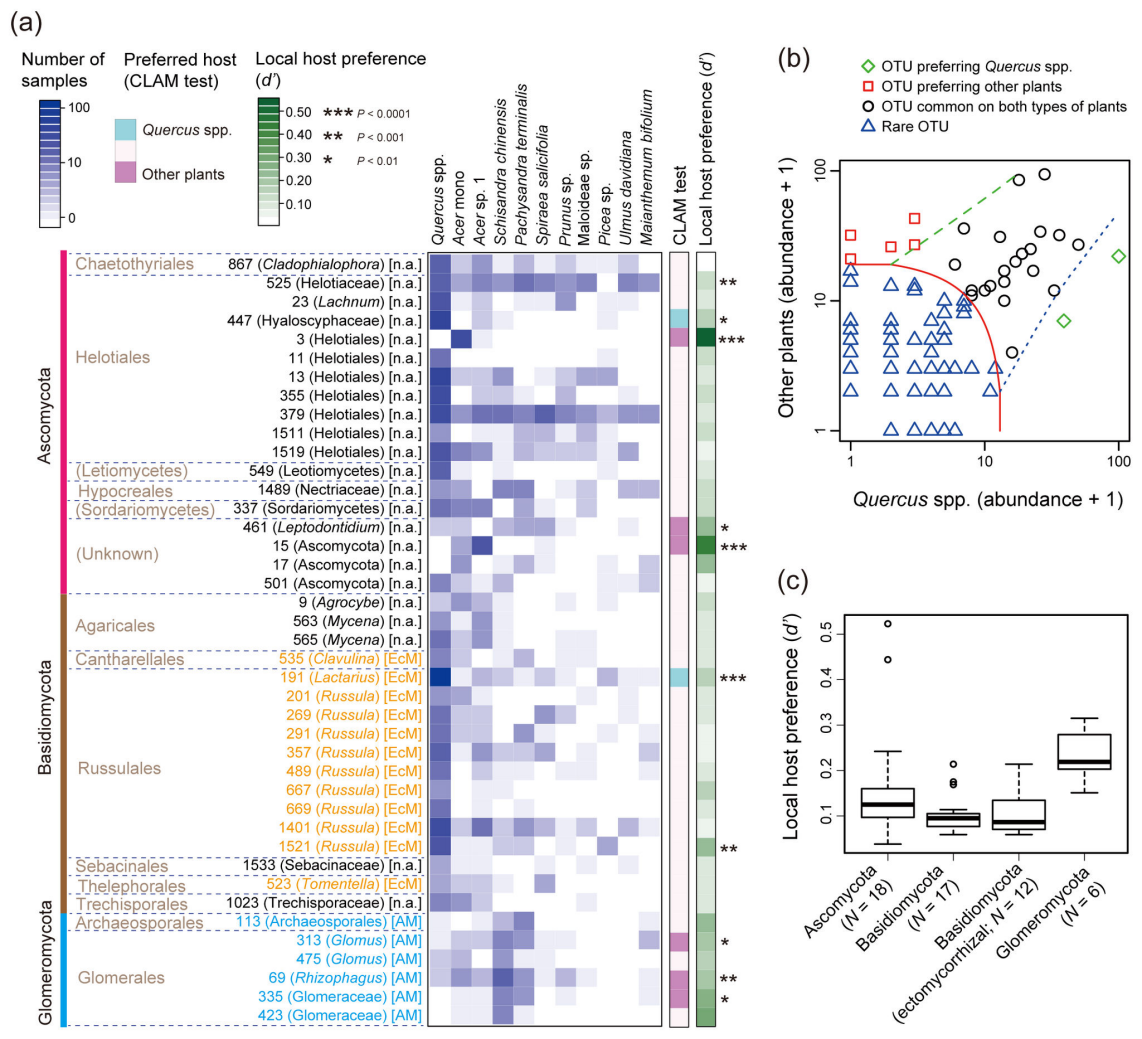
Local host preference analysis. (a) Matrix representing plant–fungal associations. A blue square represents the number of times (terminal root samples) in which a plant × fungal association was observed in the plant × fungal matrix (Appendix S4). Results of plant species with 10 or more root samples and the fungal OTUs that appeared in 10 or more root samples are shown. The fungal OTUs preferring the dominant *Quercus* species, OTUs preferring subordinate plant species, and OTUs commonly observed on both types of hosts (*sensu* [45]) are indicated by the “CLAM test” column. In addition, the *d*’ index [43] of local host preference is shown for each fungal OTU at the “Local host preference (*d*’)” column. See Appendix S2 for the results of CLAM and *d*’ measures for all the examined fungi. For each OTU, genus or family name is shown in a parenthesis and mycorrhizal type in a bracket. (b) Fungal OTUs classified by CLAM test. Note that there are perfectly overlapping symbols (Appendix S2). (c) Variation in the *d*’ measures of local host preference within taxonomic or ecological group. The *d*’ estimates of local host preferences for fungal OTUs that occurred 10 or more root samples are shown.

 Among the 278 fungal OTUs that were included in the CLAM test, an ectomycorrhizal basidiomycete fungus in the genus *Lactarius* (OTU 191) and an ascomycete fungus in the order Helotiales (OTU 447) showed statistically significant preferences for *Quercus* species (Figure 3a and b; Appendix S2). In contrast, six fungal OTUs were shown to colonize *Quercus* species less often than would be expected by chance (Figure 3b; Appendix S2). These fungi that did not select the dominant plant species as their hosts included an ascomycete fungus in the order Helotiales (OTU 3) and three common arbuscular mycorrhizal fungi (OTUs 69, 313, and 335; Figure 3a). Among them, a Helotiales fungus (OTU 3) never occurred on *Quercus* species, while it was observed in 31 root samples from plant species other than *Quercus*, especially on *A. mono* (Figure 3a). Likewise, arbuscular mycorrhizal fungi were rarely observed from *Quercus* roots, while they were detected from the roots of the woody vine *S. chinensis* and the shrub *P. terminalis* at relatively high frequencies (Figure 3a).

 In contrast to fungi with high local host preferences, 21 fungal OTUs commonly colonized the roots of both *Quercus* and subordinate plant species (Figure 3b; Appendix S2). For example, several commonly observed Helotiales fungi, OTUs 379, 525, and 1519 (Table 1), were detected from 19, 19, and 14 of the 36 plant species examined, respectively (Appendix S2). The ITS sequence of one of the Helotiales fungi perfectly matched that of a root-endophytic fungus in a warm–temperate forest that was detected in 10 of 12 plant species examined in a previous study (OTU 483 in [22]). In addition to these Helotiales fungi, many ectomycorrhizal fungi, such as OTUs 1401 and 357 in the genus *Russula*, commonly colonized not only the roots of the dominant *Quercus* species but also those of other plant species (Figure 3a). 

**Table 1:** Common fungal OTUs observed at the study site.

**OTU**		**Description**				**BLAST top-hit**			
**ID**	***N***	**Phylum**	**Order**	**Family**	**Genus**	**Description**	**E value**	**Identity**	**Accession**
379	120	Ascomycota	Helotiales			Helotiales sp.	8E-150	100%	JX243904.1
191	120	Basidiomycota	Russulales	Russulaceae	*Lactarius* *	*Lactarius quietus*	0	99%	JF273529.1
525	101	Ascomycota	Helotiales	Helotiaceae		*Rhizoscyphus* sp.	0	96%	FR837915.1
13	75	Ascomycota	Helotiales			Helotiales sp.	2E-151	100%	KC180683.1
1401	66	Basidiomycota	Russulales	Russulaceae	*Russula* *	*Russula vesca*	0	99%	AB509783.1
1519	58	Ascomycota	Helotiales			*Meliniomyces variabilis*	3E-129	96%	HQ157930.1
1521	45	Basidiomycota	Russulales	Russulaceae	*Russula* *	*Russula chloroides*	0	99%	AY061663.1
447	44	Ascomycota	Helotiales	Hyaloscyphaceae		*Albotricha* sp.	9E-115	93%	JN995639.1
69	44	Glomeromycota	Glomerales	Glomeraceae	*Rhizophagus* †	Glomeromycetes sp.	1E-132	93%	JQ272369.1
355	43	Ascomycota	Helotiales			*Meliniomyces* sp.	1E-118	94%	FN669230.1
337	42	Ascomycota				*Cephalotheca sulfurea*	4E-68	83%	AB278194.2
1489	41	Ascomycota	Hypocreales	Nectriaceae		*Neonectria* sp.	1E-157	99%	JX243941.1
867	40	Ascomycota	Chaetothyriales	Herpotrichiellaceae	*Cladophialophora*	*Cladophialophora chaetospira*	8E-160	99%	EU035403.1
23	38	Ascomycota	Helotiales	Hyaloscyphaceae	*Lachnum*	*Lachnum* sp.	2E-145	99%	JN655650.1
357	35	Basidiomycota	Russulales	Russulaceae	*Russula* *	*Russula amoenipes*	0	96%	AY061656.1
3	31	Ascomycota	Helotiales			*Botryotinia convoluta*	1E-132	98%	AF300747.1
15	31	Ascomycota				Helotiaceae sp.	2E-126	95%	JQ272370.1
269	29	Basidiomycota	Russulales	Russulaceae	*Russula* *	*Russula cerolens*	0	99%	JN681168.1
461	28	Ascomycota			*Leptodontidium*	*Leptodontium* sp.	6E-151	100%	JX244015.1
291	26	Basidiomycota	Russulales	Russulaceae	*Russula* *	*Russula quercilicis*	0	97%	JF908700.1
313	26	Glomeromycota	Glomerales	Glomeraceae	*Glomus* †	*Glomus* sp.	5E-137	93%	HE794038.1

The ID numbers of OTUs and the number of terminal root samples in which respective fungi were observed are shown. The results of molecular identification based on Claident- and manual-BLAST searches are shown for each OTU. Fungal OTUs that appeared in more than 25 root samples are shown.

* Putatively ectomycorrhizal lineages.

† Putatively arbuscular-mycorrhizal lineages.

 An additional CLAM analysis comparing fungal communities between *Quercus* spp. and common *Acer* spp. indicated that one ectomycorrhizal OTU (*Lactarius*; OTU 191) and two Helotiales OTUs (OTUs 13 and 447) preferred *Quercus* spp., while a Helotiales OTU (OTU 3) and an unidentified ascomycete OTU (OTU 15) preferentially associated with common *Acer* spp. (Figures 3a and S4a; Appendix S2). Meanwhile, 11 fungal OTUs including five Helotiales and three ectomycorrhizal (*Russula*) OTUs were commonly associated with both of the *Quercus* and common *Acer* plants (Figure S4a; Appendix S2). Another CLAM analysis comparing fungal communities between common *Acer* spp. and the remaining subordinate plants revealed that a Helotiales OTU (OTU 3) and an unidentified ascomycete OTU (OTU 15) preferred common *Acer* spp., while no fungal OTU preferentially associated with the remaining subordinate plants (Figure S4b; Appendix S2). Fourteen fungal OTUs including four ectomycorrhizal (Russulaceae), four Helotiales, and two arbuscular mycorrhizal OTUs were commonly associated with both of the common *Acer* spp. and the remaining subordinate plants (Figure S4b; Appendix S2).

 Local host preference in plant–fungal associations was also evaluated using the *d’* measure of interaction specificity based on the “plant x fungal” matrix (Appendix S4) that included 36 plant species and 278 fungal OTUs (Figure 3a and c). For the *Lactarius* and Helotiales fungi that showed significant preferences for *Quercus* spp. in the CLAM test, the *d’* estimates of local host preference were also higher than expected by chance (Figure 3a). Likewise, for the ascomycete and arbuscular mycorrhizal fungi that showed significant preferences for subordinate plant species in the CLAM test, the *d’* estimates of local host preference were significant as well (Figure 3a). The *d’* measures of local host preferences were considerably variable within taxonomic or ecological group, especially within the phylum Ascomycota (Figure 3c).

## Discussion

 In an oak-dominated cool–temperate forest in Japan, we determined the diversity of root-associated fungal communities using 454-pyrosequencing and thereby examined how fungal root symbionts were shared between dominant-oak and subordinate plant species in the plant community. The results are summarized as follows. First, the root-associated fungal community at the study site included many ectomycorrhizal basidiomycetes and arbuscular mycorrhizal glomeromycetes as well as phylogenetically and ecologically diverse clades of ascomycete fungi (Figures 1 and S1; Table 1). Second, the dominant *Quercus* species shared many ectomycorrhizal and possibly endophytic fungal taxa with subordinate plant species, while arbuscular mycorrhizal fungi were mainly hosted by subordinate plant species (Figures 2 and 3). Third, root-associated fungi in the community displayed phenotypic variation in the degree of local host preference even within taxonomic or ecological group (Figure 3). 

### Diversity and community composition of root-associated fungi

 The root-associated fungal community in the cool–temperate forest in Tomakomai was characterized by the occurrence of diverse ectomycorrhizal taxa, the coexistence of ectomycorrhizal and arbuscular mycorrhizal fungi, and the prevalence of possibly-endophytic ascomycetes in the order Helotiales.

 While the three ectomycorrhizal basidiomycete genera, *Russula*, *Lactarius*, and *Tomentella*, were commonly associated with *Quercus* and other plants species, other diverse ectomycorrhizal fungi in Basidiomycota (e.g., *Inocybe*, *Cortinarius*, *Lactarius* and *Clavulina*) and Ascomycota (e.g., *Tuber* and *Cenococcum*) occurred at the study site (Figures 1 and S2). Most of these genera are commonly found in temperate and tropical forests dominated by such plant families as Fagaceae, Pinaceae, and Dipterocarpaceae [29,32,64]. For example, *Russula*, *Lactarius*, and *Tomentella* are also common in warm–temperate forests in central Japan, where two dominant oak species (*Q. serrata* and *Q. glauca*) co-occur with diverse clades of arbuscular mycorrhizal or ericoid mycorrhizal plants such as *Ilex*, *Prunus*, and *Lyonia* [22]. 

 Although the various clades of ectomycorrhizal fungi represented the belowground plant–fungal associations in the cool–temperate forest, they were not the sole major partners of plants. That is, 8.6% of the observed plant–fungal associations involved arbuscular mycorrhizal fungi (Figure 1a). In the study forest, only *Quercus* spp. and rare *Picea* (Pinaceae) and *Carpinus* (Betulaceae) species are considered to be “ectomycorrhizal” based on the conventional classification of mycorrhizal plants [17,29], while other observed plant taxa are likely to be arbuscular mycorrhizal (or non-mycorrhizal). Given that possibly arbuscular mycorrhizal plants occur in most temperate and tropical forests dominated by species from ectomycorrhizal plant families, the coexistence of arbuscular mycorrhizal and ectomycorrhizal fungi would be a common feature of those forests ([22,65,66]; see also 67). 

 In addition to ectomycorrhizal and arbuscular mycorrhizal fungi, diverse ascomycete fungi in the order Helotiales were common at the study site. The dominance of Helotiales in root-associated fungal communities has been reported in various environments such as Arctic tundra [68] and warm–temperate forests [22]. Although the order Helotiales includes diverse fungal functional groups, such as ectomycorrhizal, saprotrophic, and endophytic species [20,21,69], several clades of fungi within the order possibly benefit their plant hosts by mineralizing organic nitrogen in the rhizosphere [21]. Intriguingly, the ITS sequence of the most commonly observed Helotiales fungus (OTU 379; Appendix S1) perfectly matched the sequence of a Helotiales root endophyte that we observed in a previous study in a warm–temperate forest [22]. Thus, Helotiales endophytes can be major participants in belowground plant–fungal associations in various types of forests, although their ecological functions to plant hosts need to be further investigated. 

### Overall structure of the belowground plant–fungal associations

 In the cool–temperate forest, an ectomycorrhizal fungus in the genus *Lactarius* and an ascomycete fungus in Helotiales preferred *Quercus* species to the remaining plant species (Figure 3). On the other hand, six fungal taxa were shown to colonize *Quercus* species less often than would be expected by chance (Figure 3b). In particular, three arbuscular mycorrhizal fungi were mainly detected from plant species such as the woody vine *S. chinensis* and the shrub *P. terminalis* (Figure 3a), and were shared among various subordinate plant species (Figure 2b). Importantly, the existence of fungi that show host preferences can result in the compartmentalization of belowground plant–fungal associations. That is, dominant and subordinate plant species interact with different subsets of the root-associated fungal community in respective “modules” of symbiotic associations (e.g., a dominant plant–ectomycorrhizal fungal module vs. a subordinate plant–arbuscular-mycorrhizal fungal module) (cf. [67]).

 Nonetheless, all of the plant species at the study site shared at least one root-associated fungal symbiont with other plant species (Figure S3), as would be expected in the presence of fungi associating with 10 or more plant species (Appendix S2). These fungi with broad host ranges include several ascomycete fungi in the orders Helotiales and Chaetothyriales (Figure 3; Appendix S2), as previously determined in a warm–temperate forest [22,70]. In addition to Helotiales and Chaetothyriales fungi, several ectomycorrhizal taxa, especially members of the genus *Russula*, displayed broad host ranges, being detected not only from the dominant *Quercus* species but also from other plant species (Figure 3). Importantly, colonization of ectomycorrhizal fungi in “non-ectomycorrhizal” plants has been reported in other studies (e.g., [71]). Our results, therefore, further indicate that the colonization of ectomycorrhizal fungi into the roots of primarily arbuscular mycorrhizal plants can be prevalent rather than exceptional in natural forests. In considering the ecological consequences of such “promiscuous” plant–fungal associations, we should keep in mind that root–hyphal physical contact does not necessarily imply that mutual ecological benefits exist between fungal symbionts and their hosts [72]. Nonetheless, the prevalence of fungi that potentially interact with both dominant ectomycorrhizal and subordinate arbuscular mycorrhizal plants suggests that simple classifications by mycorrhizal type do not fully depict the overall structure of belowground plant–fungal associations.

### Variation in local host preference

 As noted above, root-associated fungi in the cool–temperate forest displayed varying degrees of local host preference (Figure 3). Among the phyla examined, especially high variation in local host preference was observed within Ascomycota (Figure 3a and b). Helotiales in particular included fungi that had a significant preference for *Quercus* or subordinate plant species as well as generalist fungi that were commonly observed on both dominant and subordinate host species (Figure 3a). Likewise, a Helotiales OTU (OTU 3) and an unidentified ascomycete OTU (OTU 15) preferred two common *Acer* species to *Quercus* spp. or the remaining subordinate plant species, while the *Acer* species shared various clades of common ascomycete OTUs (e.g., Helotiales and Chaetothyriales) with other plant species (Figures 3a and S4; Appendix S2). 

 Variation in local host preference was also observed within ectomycorrhizal fungi. Within the family Russulaceae, many OTUs displayed broad host ranges, while a fungus closely related to *Lactarius quietus* had a narrower host range than would be expected by chance in both the CLAM and *d’* analyses (Figure 3a). Intriguingly, a fungus with the same ITS sequence was reported in a warm-temperate forest located 1000 km south of the present study site, and the fungus also displayed a significant local host preference for a deciduous oak species there, i.e., *Q. serrata* [22,70]. This suggests that a fungal species can show a consistently high host preference in different locations where plant community composition differs, while host preference itself can vary considerably among species within genera or families (Figure 3).

 Although intriguing, the high host-preference variation observed in this study should be interpreted with caution. Theoretically, the host preference of a fungus in a natural forest (i.e., its phenotype) is determined by a genotype x environment interaction between the potential host range of the fungus [35,38,39] and the composition of the local host-plant community [32,40] or abiotic soil conditions [41,42]. Therefore, we must bear in mind that the host preference phenotype of a fungal species can vary among forests that differ in plant community composition. While cross-inoculation experiments are essential for examining the potential host range of a fungus [35,38,39], observational studies of host-preference phenotypes in natural forests provide insights into plant–fungal associations that are realized in the wild based on genotype x environment interactions. The standardized index of interaction specificity (*d*’ [43];) enables us to investigate how host-preference phenotypes vary among local populations in response to local abiotic/biotic environments (e.g., plant community structure). Thus, further host-preference surveys in other types of forests (e.g., forests dominated by arbuscular mycorrhizal plants) will provide opportunities to test whether a fungal species displays a consistently high host preference under different environmental conditions or if its host preference phenotype is highly plastic. 

### Conclusions and perspectives

 In this study, we evaluated how dominant and subordinate plant species shared diverse clades of mycorrhizal and root-endophytic fungi within a local community by statistically evaluating fungal local host preference. Due to considerable variation in local host preference, each fungal functional group had different effects on the overall architecture of belowground plant–fungal associations. Arbuscular mycorrhizal fungi, for example, rarely colonized the roots of the dominant *Quercus* species, thereby making the plant–fungal associations more compartmentalized than would be expected from random host–symbiont associations. However, many ectomycorrhizal fungi and possible root endophytes were associated not only with the dominant *Quercus* species but also with the remaining plant species. Thus, the entire structure of belowground plant–fungal associations is properly described as a continuity that spans from the random sharing of fungal symbionts within a plant community to complete compartmentalization by mycorrhizal type. This complexity in belowground plant–fungal associations is of particular interest because plant species in a community also share pollinators and seed-dispersers in the aboveground environment, and the architecture of such plant–animal interaction networks can affect the stability of plant communities [1,2,7,73]. Comparisons of network structures between aboveground plant–animal interactions and belowground plant–fungal associations will help clarify the ecological mechanisms that promote the coexistence of plant species. Furthermore, the structure of a plant community itself can be an important determinant of root-associated fungal community composition in a local forest [74,75] (cf. [32,40]). Community ecological studies that simultaneously target the entire plant and root-associated fungal communities are essential to understand the inter-dependence of those communities’ dynamics.

## Supporting Information

Figure S1 **Summary of the pyrosequencing.** (a) Rarefaction curve of OTUs in each root sample against the number of pyrosequencing reads excluding singletons. (b) Composition of host plant species identified by chloroplast *rbcL* sequences (*N* = 577 root samples).
(PDF)

Figure S2 **Community composition of root-associated fungi.** (a) Phylum-level composition of fungal OTUs observed in root samples. Asterisk indicate the fungi whose phylum level taxonomy is unsettled. (b) Order-level composition of fungal OTUs. Asterisk indicate the fungi whose order level taxonomy is unsettled. (c) Genus-level composition of fungal OTUs.
(PDF)

Figure S3 **Sharing of fungal OTUs among plant species in the community (all fungal OTUs).** The number of fungal OTUs shared among plant species is shown. The line thickness is proportional to the number of fungal OTUs shared between each pair of plant species. The size of circles roughly represents the composition of plant species in the samples (Figure S1b).
(PDF)

Figure S4 **Fungal OTUs classified by CLAM test (supplementary tests).** (a) Common *Acer* spp. vs. *Quercus* spp.. OTUs preferring common *Acer* spp., those preferring *Quercus* spp., OTUs common on both types of plants, and rare OTUs were indicated separately. Note that there are perfectly overlapping symbols (Appendix S2). (b) Common *Acer* spp. vs. the remaining subordinate species. OTUs preferring common *Acer* spp., those preferring the remaining subordinate plants (i.e., plant species other than *Quercus* spp. and common *Acer* spp.), OTUs common on both types of plants, and rare OTUs were indicated separately. Note that no fungal OTU was classified as that preferring the remaining subordinate plants.
(PDF)

Appendix S1 **Fungal OTU sequences in FASTA format.**
(TXT)

Appendix S2 **Fungal OTUs detected from the root samples.**
(XLSX)

Appendix S3 **Matrix representing the presence/absence of fungal OTUs in each root sample.**
(XLSX)

Appendix S4 **Matrix representing the symbiosis of plant species and fungal OTUs.**
(XLSX)
