# Supplementary figures and images for: Sharing of Diverse Mycorrhizal and Root-Endophytic Fungi among Plant Species in an Oak-Dominated Cool–Temperate Forest
Source: PLoS One. 2013 Oct 21;8(10):e78248. doi: 10.1371/journal.pone.0078248 (PMC3824041; doi:10.1371/journal.pone.0078248)

(a)

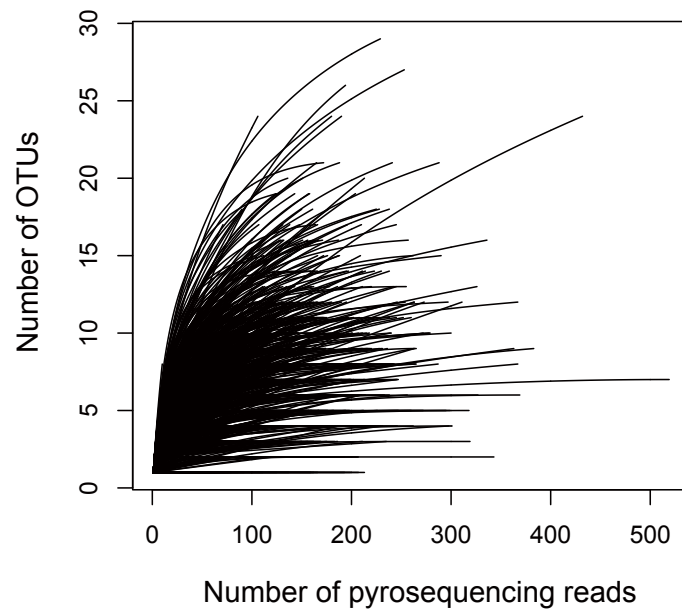

(b)

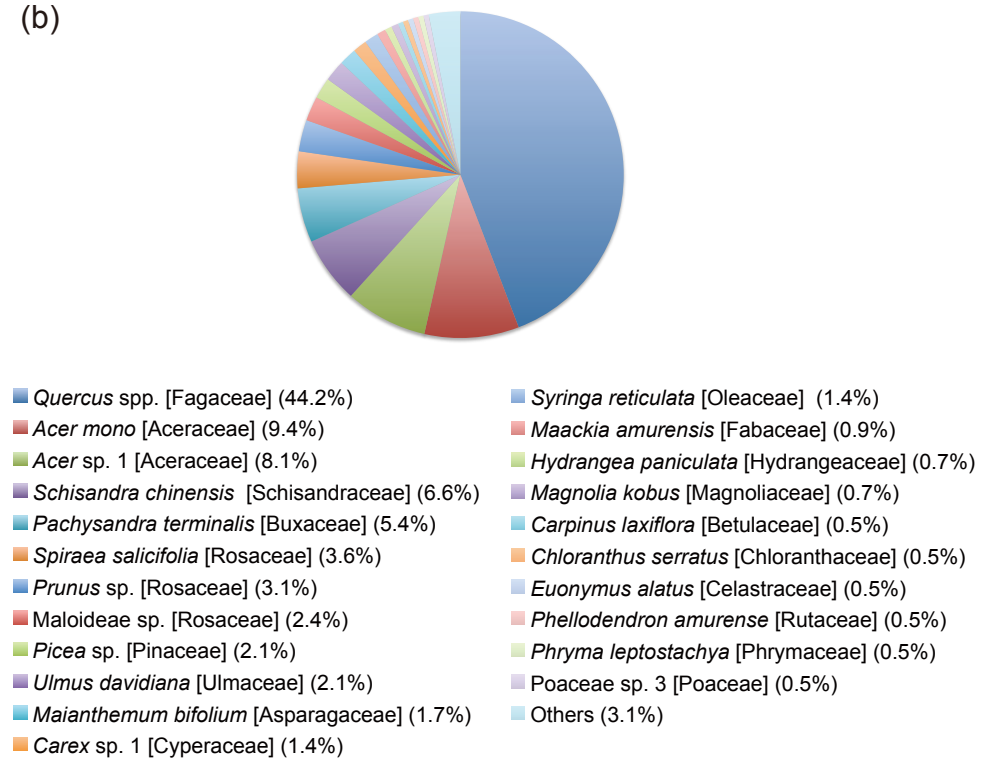

Fig. S1

Supplement: Figure S1 — Summary of the pyrosequencing. (a) Rarefaction curve of OTUs in each root sample against the number of pyrosequencing reads excluding singletons. (b) Composition of host plant species identified by chloroplast rbcL sequences (N = 577 root samples). (PDF) [file pone.0078248.s001.pdf]

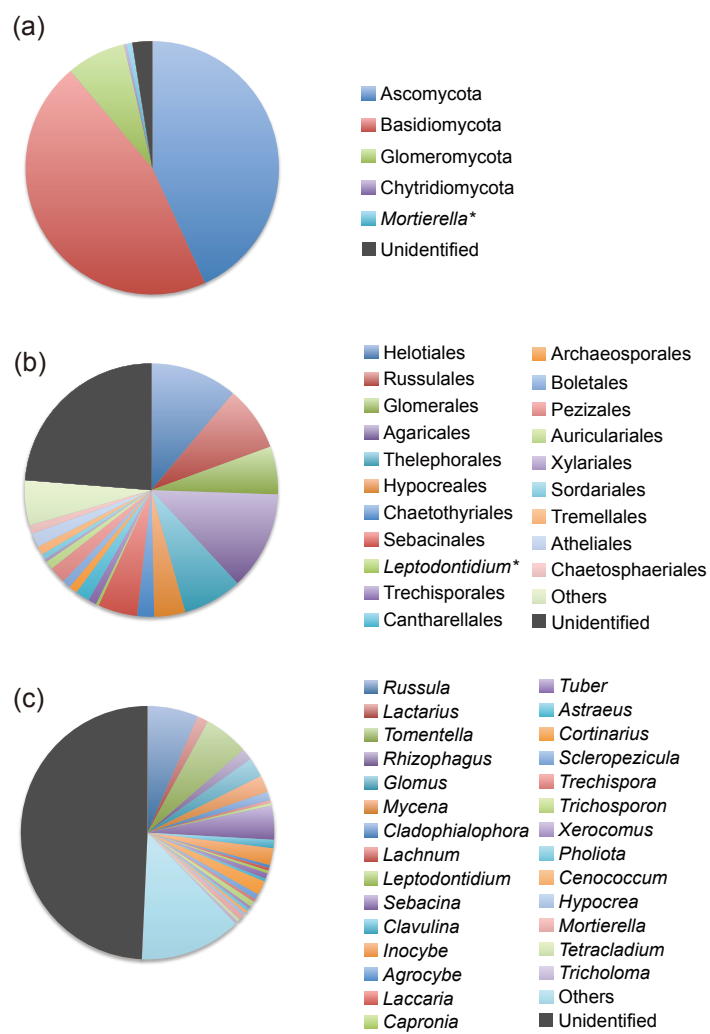

Fig. S2

Supplement: Figure S2 — Community composition of root-associated fungi. (a) Phylum-level composition of fungal OTUs observed in root samples. Asterisk indicate the fungi whose phylum level taxonomy is unsettled. (b) Order-level composition of fungal OTUs. Asterisk indicate the fungi whose order level taxonomy is unsettled. (c) Genus-level composition of fungal OTUs. (PDF) [file pone.0078248.s002.pdf]

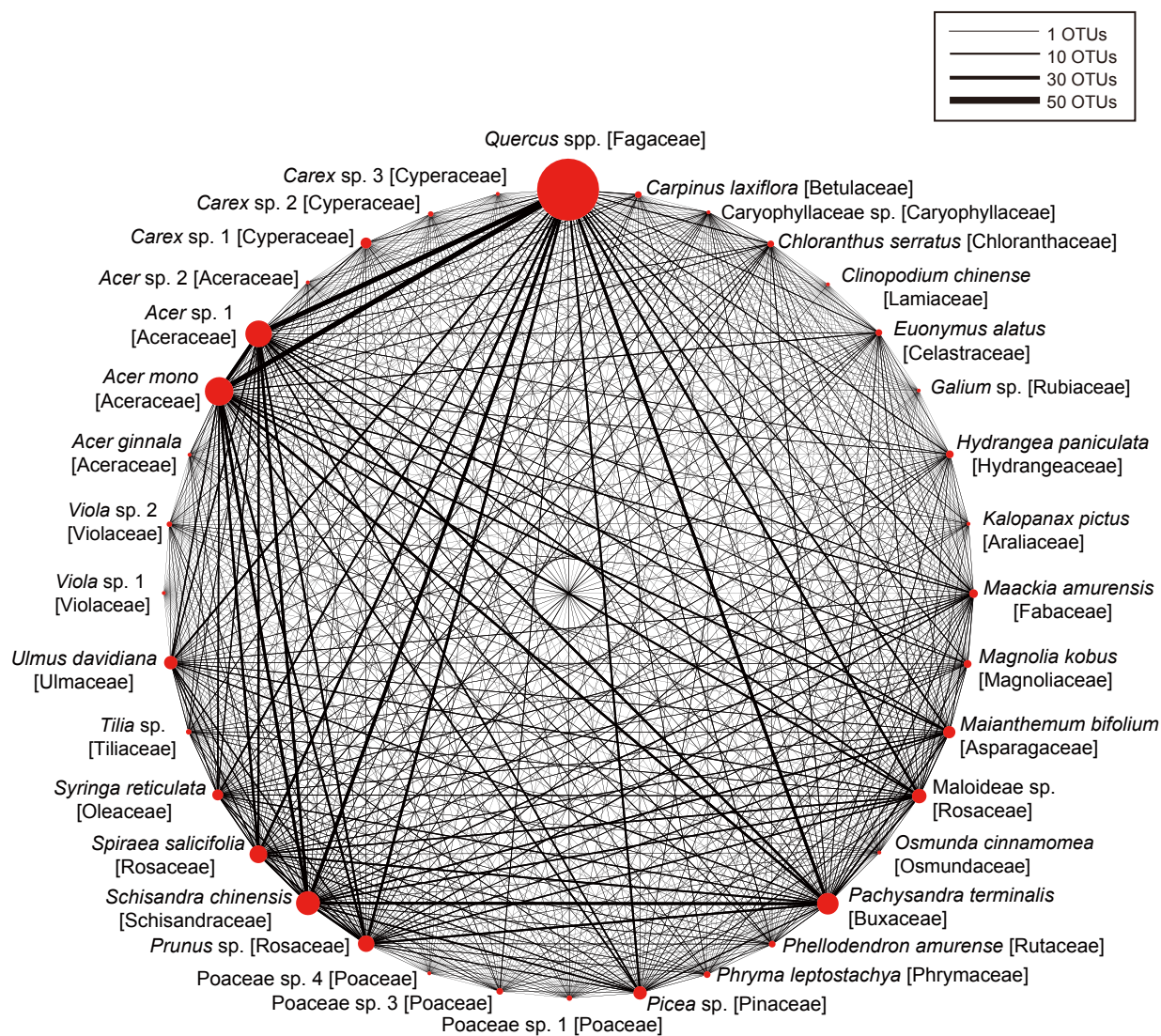

Fig. S3

Supplement: Figure S3 — Sharing of fungal OTUs among plant species in the community (all fungal OTUs). The number of fungal OTUs shared among plant species is shown. The line thickness is proportional to the number of fungal OTUs shared between each pair of plant species. The size of circles roughly represents the composition of plant species in the samples (Figure S1b). (PDF) [file pone.0078248.s003.pdf]

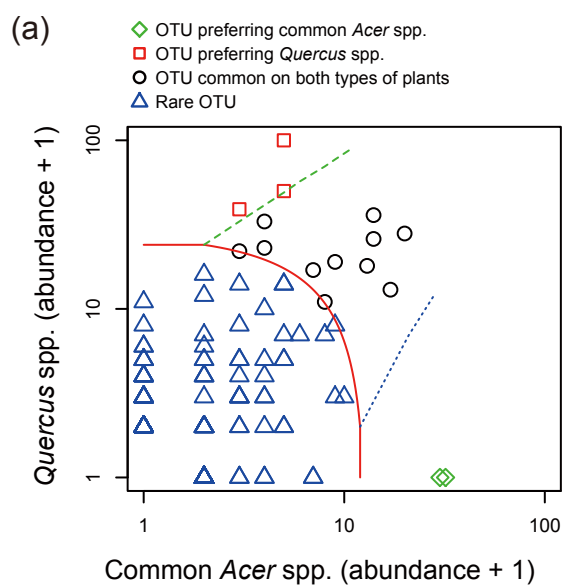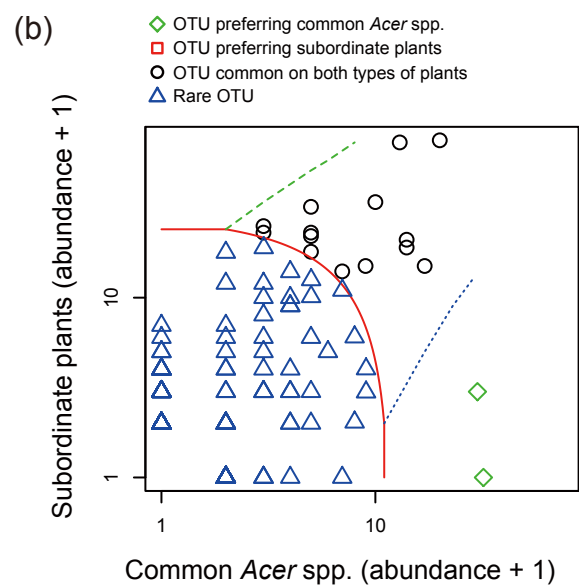

Fig. S4

Supplement: Figure S4 — Fungal OTUs classified by CLAM test (supplementary tests). (a) Common Acer spp. vs. Quercus spp.. OTUs preferring common Acer spp., those preferring Quercus spp., OTUs common on both types of plants, and rare OTUs were indicated separately. Note that there are perfectly overlapping symbols (Appendix S2). (b) Common Acer spp. vs. the remaining subordinate species. OTUs preferring common Acer spp., those preferring the remaining subordinate plants (i.e., plant species other than Quercus spp. and common Acer spp.), OTUs common on both types of plants, and rare OTUs were indicated separately. Note that no fungal OTU was classified as that preferring the remaining subordinate plants. (PDF) [file pone.0078248.s004.pdf]
